# Topoisomerase Inhibitors and PIM1 Kinase Inhibitors Improve Gene Editing Efficiency Mediated by CRISPR-Cas9 and Homology-Directed Repair

**DOI:** 10.3390/molecules29122890

**Published:** 2024-06-18

**Authors:** Ying Yun, Min Wang, Shimeng Guo, Xin Xie

**Affiliations:** 1 School of Pharmaceutical Science and Technology, Hangzhou Institute for Advanced Study, University of Chinese Academy of Sciences, Hangzhou 310024, China; yunying@ucas.ac.cn; 2 State Key Laboratory of Drug Research, National Center for Drug Screening, Shanghai Institute of Materia Medica, Chinese Academy of Sciences, Shanghai 201203, China; mwang@simm.ac.cn (M.W.); gshimeng@simm.ac.cn (S.G.); 3 Shandong Laboratory of Yantai Drug Discovery, Bohai Rim Advanced Research Institute for Drug Discovery, Yantai 264117, China

**Keywords:** CRISPR-CAS9, gene editing, homology-directed repair, knock-in

## Abstract

The CRISPR-Cas9 system has emerged as the most prevalent gene editing technology due to its simplicity, high efficiency, and low cost. However, the homology-directed repair (HDR)-mediated gene knock-in in this system suffers from low efficiency, which limits its application in animal model preparation, gene therapy, and agricultural genetic improvement. Here, we report the design and optimization of a simple and efficient reporter-based assay to visualize and quantify HDR efficiency. Through random screening of a small molecule compound library, two groups of compounds, including the topoisomerase inhibitors and PIM1 kinase inhibitors, have been identified to promote HDR. Two representative compounds, etoposide and quercetagetin, also significantly enhance the efficiency of CRISPR-Cas9 and HDR-mediated gene knock-in in mouse embryos. Our study not only provides an assay to screen compounds that may facilitate HDR but also identifies useful tool compounds to facilitate the construction of genetically modified animal models with the CRISPR-Cas9 system.

## 1. Introduction

Gene editing is a precise genetic engineering technology that enables targeted modification of specific genes within an organism’s genome, with significant potential for genetic research and gene therapy [1]. While ZFN and TALEN are common gene editing tools, their cumbersome operation, high cost, and limited scalability have restricted their use. In contrast, CRISPR-Cas9 is currently the simplest, most affordable, and widely used gene editing technology; it has been hailed as one of the greatest biotechnological discoveries of this century [2,3]. CRISPR-Cas9 has revolutionized the ways of genetic studies, which facilitate disease research and accelerate drug discovery. It also has a profound impact on microbial-based industrial production and food crop development [4,5].

The type II system from Streptococcus pyogenes, which comprises the sgRNA for target sequence identification and the Cas9 protease for target sequence cleavage, is the most utilized CRISPR-Cas9 system. The Cas9-sgRNA complex induces DNA double-strand breaks (DSBs), which are repaired by different pathways, subsequently resulting in directional editing of DNA [6]. In mammalian cells, DSBs can be repaired via non-homologous end joining (NHEJ) or homology-directed repair (HDR). NHEJ offers high efficiency but poor specificity, leading to random insertion or deletion of DNA, whereas HDR utilizes homologous sequences as donor templates to achieve accurate base pair insertion, deletion, or mutation with good fidelity albeit low efficiency [7,8,9].

Scientists have explored various approaches to enhance HDR efficiency. Optimization of sgRNA and Cas9 proteins has been shown to affect the ratio of HDR and NHEJ [10]. Increasing the frequency of HDR can also be achieved through optimizing DNA donor templates and increasing their local concentration at double-strand break sites [11]. Single-strand oligonucleotide templates are considered to be more efficient for HDR than double-strand DNA templates, and the higher efficiency is thought to be associated with greater numbers of nearby DNA donor templates [12]. Moreover, a number of studies have utilized small molecule compounds to modulate the key proteins involved in DNA repair pathways. For instance, Scr7 is a DNA ligase IV inhibitor and can significantly enhance the efficiency of HDR by inhibiting NHEJ [13]. RS1 activates Rad51 and promotes its binding with DNA, thereby prolonging the length of protein–DNA complexes and improving HDR efficiency [14]. Additionally, cell cycle progression plays a crucial role in determining the choice of DNA repair pathway; NHEJ can occur at any stage of cell division, while HDR primarily occurs during G2 and S phases. Therefore, blocking cell cycle progression using compounds such as vinblastine, aphidicolin, or nocodazole can improve HDR efficiency [15,16]. Furthermore, chromatin density also affects HDR efficiency; HDAC inhibitors have been found to increase HDR efficiency by increasing chromatin openness [17].

Although strategies exist to enhance HDR efficiency, those that require system transformation may lead to cumbersome experimentation and increased complexity of the tools, which could pose challenges for future virus packaging and transmission [18]. Additionally, reported small molecule compounds have yielded inconsistent results across different experimental systems, and their potential damage to cell DNA must also be considered [19,20,21]. Therefore, it is valuable to identify more small molecule compounds with high efficacy but low cytotoxicity. In our previous gene editing work, we encountered challenges related to the low efficiency of HDR using the CRISPR-Cas9 system, resulting in only approximately 5% successful homologous recombination events [22]. To tackle this issue, we developed a reporter system to quantify HDR efficiency and used it to screen compounds that might promote HDR in cells. We identified that both topoisomerase inhibitors and PIM1 kinase inhibitors not only enhanced HDR in the screening system but also HDR in mouse zygotes, confirming their value in real-world applications.

## 2. Results

### 2.1. Design of the In Vitro GFP Reporter-Based HDR Model

**Figure 1:**
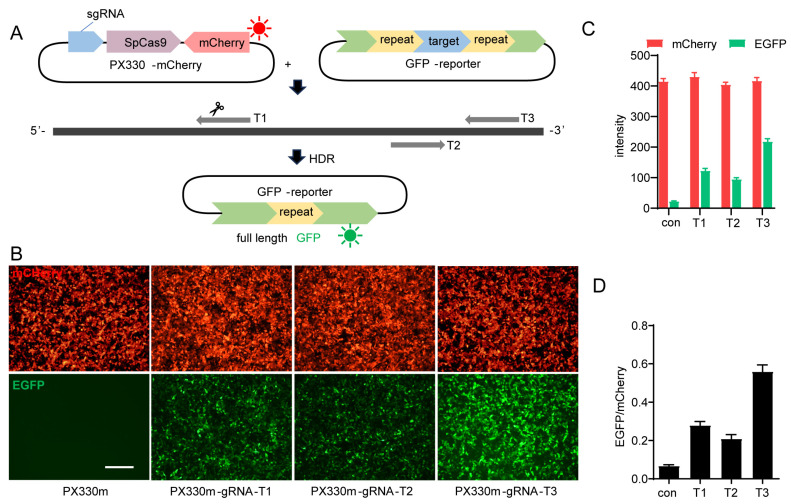
Establishment of GFP reporter-based HDR model for chemical screening. (**A**) Design of GFP reporter-based HDR model. The sgRNA was inserted into PX330-mCherry plasmid, which also expresses SpCas9 and mCherry. The target DNA of the sgRNA was flanked by repeated sequences of GFP in a GFP reporter plasmid. Cleavage of the target DNA by Cas9 protein under the guidance of the sgRNA (T1, T2, and T3 represent 3 tested sgRNAs) leads to HDR, which removes the target sequence and one of the repeated sequences to form the full-length functional GFP. (**B**,**C**) Representative images (**B**) and statistical analysis (**C**) of the editing efficiency induced by three sgRNAs targeting mouse TGR5 gene (GenBank Accession No. NM_174985.2, 200–350). HEK293T cells seeded onto 96-well plate (2 × 10^4^ cells/well) were transfected with GFP reporter (100 ng/well) with either PX330-mCherry (PX330m, 100 ng/well) or PX330-mCherry carrying gRNAs (PX330m-gRNA-T1~T3, 100 ng/well) with Fugene; images were taken 24 h later. (**D**) The fluorescence intensity and the intensity ratio of EGFP to mCherry in (**B**). Scale bars represent 100 μm. Data are means ± SEM (*n* = 3).

To screen compounds that enhance gene editing efficiency, we need an intuitive and quantifiable assay. Therefore, we developed a GFP reporter system based on homologous recombination induced by CRISPR-Cas9-mediated double-strand break (DSB) in the target DNA sequence. We modified the GFP sequence by introducing two repeated sequences flanking a target DNA sequence (GenBank Accession No. NM_174985.2, 200–350), which can be targeted by various sgRNAs (T1, T2, T3). The Cas9 nuclease can then induce DSB in the target DNA sequence, and the two repeated sequences within GFP serve as homologous arms that facilitate homology-directed repair (HDR) of the DNA. Consequently, this will result in the removal of the inserted target sequence and one of the repeated sequences to generate a full-length and active GFP protein capable of emitting green fluorescence, which represents gene editing and HDR efficiency. Simultaneously, sgRNAs were inserted in the PX330-mCherry vector (PX330m), which contained the Cas9 and mCherry sequences, the latter allowing for easy monitoring of both sgRNA and Cas9 protein expression by observing the red fluorescence signal (Figure 1A).

The GFP reporter vector containing the target sequence and px330m vector harboring Cas9 and different sgRNA sequences were co-transfected into HEK293T cells. The transfection efficiencies were similar across the combinations since the expression levels of mCherry were similar (Figure 1B,C). However, different sgRNAs led to different HDR efficiency, with sgRNA-T3 being the most effective and sgRNA-T2 being the least efficient (Figure 1B,C). These results demonstrate that this detection model enables rapid and convenient assessment of sgRNA cleavage and HDR efficiency.

### 2.2. Optimization of GFP-Report Detection Model and Compound Screening

**Figure 2:**
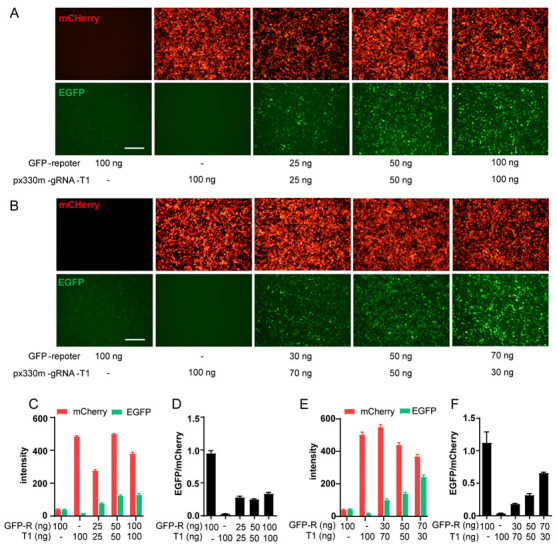
Optimization of GFP reporter-based HDR model. (**A**,**B**) Optimization of the absolute concentration (**A**) and volumetric concentration proportion (**B**) of GFP reporter plasmid and PX330m-gRNA-T1 plasmid. HEK293T cells seeded onto 96-well plate (2 × 10^4^ cells/well) were transfected with indicated amount of plasmid combination/well with Fugene; images were taken 24 h later. Scale bars represent 100 μm. (**C**,**D**) Statistical analysis of fluorescence intensity and EGFP/mCherry ratio in (**A**). (**E**,**F**) Statistical analysis of fluorescence intensity and EGFP/mCherry ratio in (**B**). Scale bars represent 100 μm. Data are means ± SEM (*n* = 3).

To ensure a suitable detection window for compound screening, sgRNA-T1, which leads to a moderate level of HDR and GFP signal, was selected for subsequent optimization and screening (Figure 1B,D). The transfection of the GFP reporter alone resulted in a low level of spontaneous HDR, as indicated by a minimal green fluorescence level and a clear background in the red channel. Transfection with PX330m-T1 alone demonstrated normal transfection efficiency, with approximately 90% of cells being red fluorescence-positive while leaving a clear background in the green channel (Figure 2A,B). We then investigated the optimal plasmids amount and ratio. Increasing the amount of both plasmids simultaneously increased the expression of red and green fluorescence. However, when both plasmids reached 100 ng, the brightness of red fluorescence decreased compared to that of 50 ng, indicating a potential exceeding of the protein synthesis capacity within the cells (Figure 2A,C,D). When the total amount of plasmid remains constant, an increase in the fraction of the GFP-reporter vector leads to enhanced green fluorescence and weakened red fluorescence (Figure 2B,E,F). Taken together, 50 + 50 ng plasmids per well into 2 × 10^4^ cells yielded stable transfection and protein expression efficiency, as well as a moderate HDR level.

**Figure 3:**
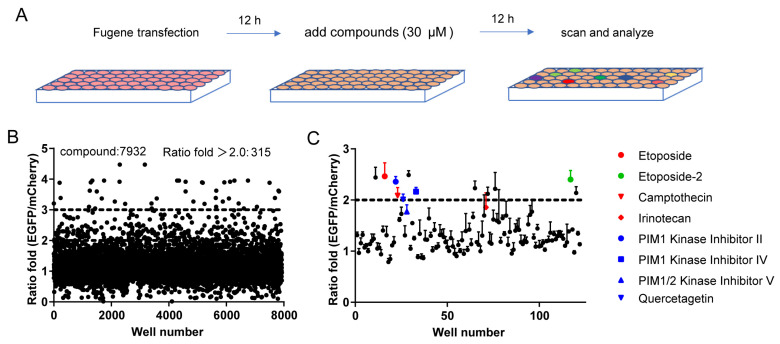
Compound screening. (**A**) Diagram of compound screening. Transient transfection was performed on 2 × 10^4^ HEK293T cells with 50 ng of GFP reporter plus 50 ng of PX330m-gRNA-T1 per well with Fugene. Compounds (30 μM) were added 12 h after transfection. Another 12 h later, fluorescence images were taken and analyzed. (**B**) Initial screening results of 7932 compounds. The EGFP/mCherry ratio is calculated for each compound and then normalized to that of DMSO control. (**C**) Repeated screening of 122 candidate compounds. Data are means ± SEM (*n* = 3).

So, this condition was used to screen 7932 compounds containing many old drugs and pathway regulators from the Chinese National Compound Library at a concentration of 30 μM. Compounds were added 12 h after transfection; then, after another 12 h, red and green fluorescence images were captured using the operetta CLS™ high-content imaging analysis system (Figure 3A). The ratio between green and red fluorescence intensity was calculated and then normalized to DMSO control (Figure 3B). Eighty compounds that showed >three-fold increase in the green/red ratio from the primary screen were selected for the secondary screen in triplicates (Figure 3C). Interestingly, several topoisomerase inhibitors and PIM1 kinase inhibitors were identified as the most effective compounds in promoting HDR (Figure 3C).

### 2.3. Enhanced Efficiency of HDR in the Reporter System by Topoisomerase Inhibitors and PIM1 Kinase Inhibitors

**Figure 4:**
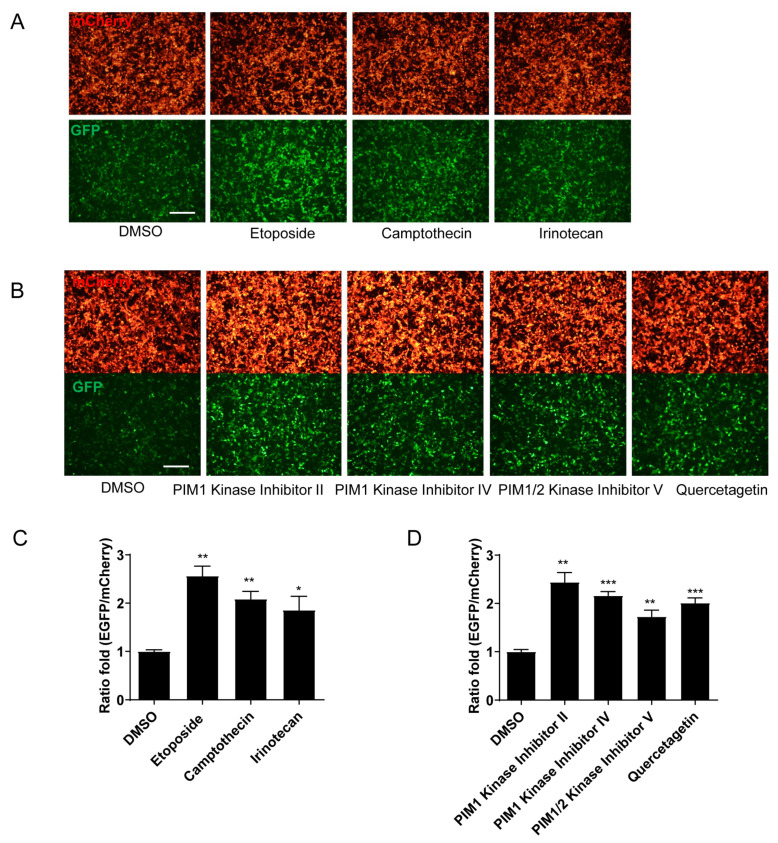
Topoisomerase inhibitors and PIM1 kinase inhibitors enhance HDR efficiency in GFP reporter-based model. (**A**,**B**) Representative images (**A**) and statistical analysis (**B**) of HDR efficiency in reporter cells treated with three different topoisomerase inhibitors (30 μM). (**C**,**D**) Representative images (**C**) and statistical analysis (**D**) of HDR efficiency in reporter cells treated with four different PIM1 kinase inhibitors (30 μM). Scale bars represent 100 μm. Data are means ± SEM (*n* = 3). Two-tailed Student’s *t*-tests were performed. * *p* < 0.05; ** *p* < 0.01; *** *p* < 0.001 versus DMSO control.

Compared to the DMSO control group, treatment with topoisomerase inhibitors, including etoposide, camptothecin, and irinotecan (all at 30 μM), significantly enhanced green fluorescence intensity while maintaining the expression of the red fluorescent protein and, thus, increased the ratio of green/red fluorescence intensity (Figure 4A,C). PIM1 kinase inhibitors were another class of compounds that significantly enhanced the ratio of green/red fluorescence intensity. Different from the topoisomerase inhibitors, PIM1 kinase inhibitors II, IV, and V, except quercetagetin, enhanced both the green and red fluorescent protein levels, while the upregulation of green fluorescence level was greater, as demonstrated by the increased green/red ratio (Figure 4B,D). These phenomena indicate that the two groups of compounds might have different mechanisms for regulating HDR efficiency.

### 2.4. Etoposide and Quercetagetin Enhance CRISPR/HDR-Mediated Gene Knock-in in Mouse Zygotes

**Figure 5:**
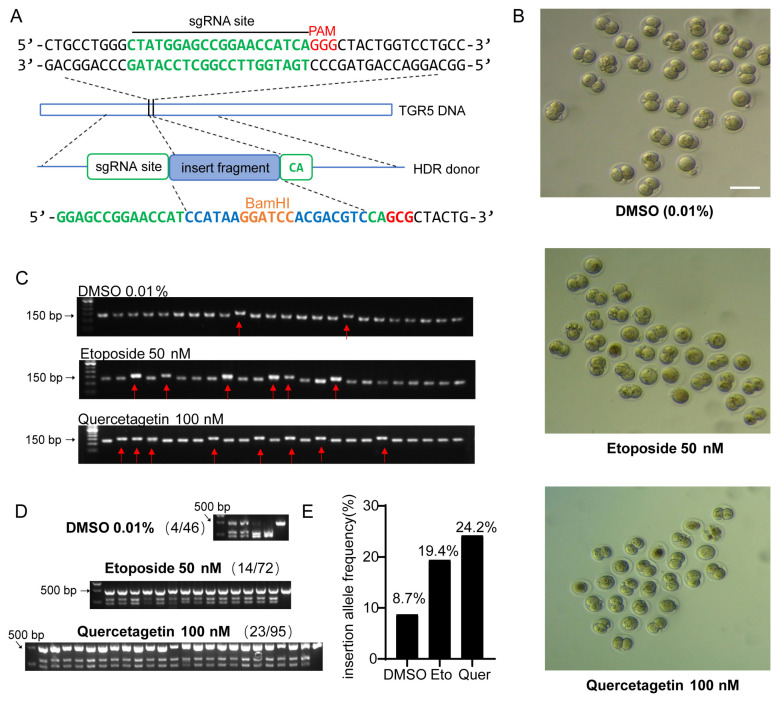
Topoisomerase inhibitor and PIM1 kinase inhibitor enhance the efficiency of CRISPR/HDR-mediated knock-in in mouse zygotes. (**A**) Schematic diagram of the precise BamH1 site insertion in mouse TGR5 gene. gRNA target site and PAM sequence are shown in green and red, respectively. The donor ssODN contains a 20 bp insert fragment (highlighted in blue) that includes BamH1 (highlighted in orange). (**B**) Early development of embryos after microinjection of the CRISPR/HDR system and cultured in vitro in the presence of etoposide (50 nM) or quercetagetin (100 nM) for 24 h. Scale bars represent 250 μm. (**C**) Representative gel images showing 20 bp insertion in designated site in embryonic genome 24 h after microinjection and drug treatment. Each lane represents an embryo. The first lane is DNA marker, and the red arrows indicate embryos with 20 bp insertion. (**D**) BamH1 cleavage of the insertion. Samples with 20 bp insertion were selected, and 480 bp PCR products containing the insert fragment were treated with BamH1, and successful cleavage of the 480 bp fragment to 223 bp and 257 bp were observed from all selected samples. The first lane is DNA marker, and the last lane is sample from WT mice. (**E**) Statistical analysis of the insertion efficiency.

We then tested whether these compounds could be used to enhance the efficiency of HDR-mediated gene knock-in in mouse zygotes. We intended to insert a 20 bp sequence containing the BamHI site, which could be used to detect the insertion behind the 233rd base of the TGR5 gene CDS region. A donor sequence was designed to contain the 20 bp insertion within the sgRNA-T1 target sequence, flanked by 50 bp homologous arms on both sides. Synonymous mutation was also introduced in the PAM region to prevent constant cleavage by Cas9 after repair (Figure 5A).

The zygotes were injected with SgRNA-T1, Cas9 protein, and HDR donor via microinjection into the cytoplasm. Then, the eggs were cultured in vitro in a medium containing vehicle, etoposide (50 nM), or quercetagetin (100 nM). Twenty-four hours later, the proportion of embryos at the two-cell stage was consistent across all three groups (80–90%), indicating that the compound treatment did not affect early embryo development (Figure 5B). Single embryo DNA samples were collected and prepared for PCR amplification of the targeted insertion region; the 126 bp PCR product indicated no insertion, while the 146 bp product indicated successful insertion of the 20 bp (Figure 5C). The 146 bp PCR products were collected and further validated by BamH1 digestion; all showed clear cleavage by BamH1, indicating the correct insertion (Figure 5D). In the vehicle group, only 4 out of 46 embryos showed correct insertion, while 14 out of 72 embryos and 23 out of 95 embryos showed correct insertion in the etoposide or quercetagetin-treated groups, respectively (Figure 5D,E). Our results demonstrate that both etoposide and quercetagetin significantly enhance CRSPR/Cas9 and HDR-mediated gene editing, resulting in a two- to three-fold increase in efficiency (Figure 5E).

## 3. Discussion

Here, we discovered that topoisomerase inhibitors and PIM1 kinase inhibitors could promote HDR and greatly enhance the efficiency of CRISPR-Cas9 and HDR-mediated gene knock-in in mouse embryos. Topoisomerases participate in the regulation of the supercoil structure and correct the number of DNA links by inducing transient DNA strand break, rewind, and re-connection [23]. In mammals, there are two main types of topoisomerases, topoisomerase I and Topoisomerase II. Topoisomerase I mainly forms short-term single-strand cleavage to allow for passing of the other DNA strand through the break or the rotation of downstream DNA duplex and then resealing the broken strand [24]. Topoisomerase II can induce DNA double-strand break, allow for passage of another duplex DNA, and, thus, adjust the topological state of DNA [25]. A variety of compounds have been discovered as topoisomerase inhibitors; however, different from traditional inhibitors, their mechanism is not to inhibit the enzyme activity but to affect the equilibrium reaction between enzyme and DNA complex, prolonging and stabilizing the formation of DNA breakage [26,27]. The gradual accumulation of DNA damage leads to cytotoxicity and cell cycle arrest in the G2 phase. Therefore, topoisomerase inhibitors have been extensively studied as antitumor drugs [27].

Applying topoisomerase inhibitors to the CRISPR-Cas9 system stabilizes DNA strand breaks and forces the cells to stay in the S/G2 phase, all of which may facilitate HDR when a large number of repair templates are provided [28,29]. This was observed in both the HDR-reporter system and mouse embryos. Topoisomerase I inhibitors stabilize single-strand breaks, while topoisomerase II inhibitors stabilize double-strand breaks. This explains why etoposide, a topoisomerase II inhibitor, is more effective than the other two topoisomerase I inhibitors in our HDR-reporter system. Although these compounds are cytotoxic when applied in high concentrations and long duration, short time usage at low doses does not affect the early development of embryos.

PIM1 kinase, a member of the PIM gene family encoding serine/threonine kinases, is a proto-oncogene involved in cell cycle regulation and tumor development. Its inhibitors are being researched as potential antitumor drugs [30,31]. PIM1 can phosphorylate many cell factors involved in apoptosis and cell cycle regulation, including c-Myc, Bad, Bcl-2, Cdc25c, NFATcl, etc., which play important roles in tumor generation [32,33]. PIM1 kinase has also been reported to affect the expression of some proteins related to cell cycle regulation, such as CDK-interacting protein/kinase inhibition protein, nuclear mitotic apparatus protein, cell division cyclin, etc. [34]. However, the precise mechanism of action of PIM1 kinase and the associated physiological and pharmacological effects of PIM1 kinase inhibitors remain unclear. In our HDR-reporter system, it is possible that PIM1 kinase inhibitors may modulate cell cycle progression or directly impact proteins involved in DNA repair to enhance HDR efficiency. Further investigation is required to elucidate the specific mechanism.

In summary, we have developed a facile and rapid in vitro system to evaluate HDR efficiency after CRISPR-Cas9-mediated DNA cleavage and identified that topoisomerase inhibitors and PIM1 kinase inhibitors could enhance HDR efficiency. Two representative compounds, etoposide and quercetagetin, also significantly enhance the efficiency of CRISPR-Cas9 and HDR-mediated gene knock-in. Although the precise underlying mechanisms require further investigation, these compounds can be used to facilitate gene knock-in in mouse embryos and increase the success rate in generating useful animal models.

## 4. Materials and Methods

### 4.1. Plasmid Construction

GFP-reporter vector: The full-length EGFP genes were amplified from the pEGFP-N1 vector and sub-cloned in the pcDNA3.1 vector to optimize the restriction site. In-Fusion HD Cloning Kit (Takara, Shiga, Japan) was used to repeatedly insert the 266–470 base sequence of EGFP after the 470 bases of EGFP, and then a synthetic 24 bp sequence containing BamH I and Hind III sites was added into the two repeats to facilitate the subsequent construction of the target sequence of sgRNA. Sequences of vectors were verified by DNA sequencing.

The designed sgRNA and target sequences (Table 1) were constructed into the PX330-mCherry Vector (Addgene, Watertown, MA, USA) and GFP-Reporter vector by adding the corresponding sticky end of the restriction site through sequence synthesis.

### 4.2. Cell Culture and Transfections

The HEK293T cell line was provided by Meiluncell, Ltd. (Dalian, China). HEK293T cells were cultured in DMEM supplemented with 10% (*v*/*v*) fetal bovine serum and incubated in 5% CO_2_ at 37 °C. For transient transfection, approximately 4 × 10^4^ cells were seeded into 96-well plates and incubated for 24 h. Plasmids and fugene (Promega, Madison, WI, USA) in a ratio of 1:3 were added into the cell culture medium and incubated for 24–48 h. Fluorescence images were taken with the Olympus IX73 inverted microscope (Olympus Corporation, Tokyo, Japan) and processed by Photoshop.

### 4.3. Compound Screening

A total of 4 × 10^4^ HEK293T cells were seeded into 96-well plates and incubated for 24 h; Plasmids (50 ng of GFP reporter plus 50 ng of PX330m-gRNA-T1 per well) and fugene (0.3 μL per well) in a ratio of 1:3 were added into the cell culture medium. A total of 7932 compounds from the national new drug screening center of Shanghai Institute of Materia Medica, Chinese Academy of Sciences (30 μM) were added 12 h after transfection. Another 12 h later, fluorescence images were taken by Opera Phenix Plus High-Content Screening System (Revvity, Waltham, MA, USA). The ratio fold was calculated as compound (EGFP intensity/mCherry intensity)/DMSO (EGFP intensity/mCherry intensity).

### 4.4. Embryonic Genomic DNA Extraction and PCR Validation

C57BL/6j was purchased from SLAC Laboratory Animal (Shanghai, China). Animals maintained under a 12 h light/dark cycle with a normal chow diet and free access to water.

Targeting sgRNA was transcribed in vitro using MEGA shortscript T7 kit (Thermo Fisher Scientific, Waltham, MA, USA) and purified according to the standard protocol by phenol: chloroform extraction and ethanol precipitation, and then dissolved in DNase/RNase-free water (Life Technologies, Gaithersburg, MD, USA). The gene-specific HDR-detect single-stranded donor oligodeoxynucleotide (ssODN) was designed to contain a 20 bp exogenous sequence, including BamH1 restriction site flanked on each side by 50–60 bases homologous to the sequence adjoining the double-strand break (DSB). Targeting sgRNA, ssODN, and Cas9 protein (Invitrogen, Carlsbad, CA, USA were diluted in microinjection buffer (10 mM Tris, 0.1 mM EDTA, pH 7.2) to the indicated working concentrations (100 ng/μL, 200 ng/μL, 15 μM, respectively).

Approximately 300 embryos were isolated from 16 C57BL/6j female mice (6–8 weeks old), which were super-ovulated by intraperitoneally injecting with PMSG (5–6 IU) (pregnant mare serum gonadotropin) and hCG (6–7 IU) (human choionic gonadotophin), and then mated to C57BL/6j male mice. The zygotes were harvested from oviducts and received the injection. Injected zygotes were cultured in vitro in a medium containing vehicle (0.01% DMSO), etoposide (50 nM), or quercetagetin (100 nM) for 24 h. Zygotes were then collected in 5 μL G1 buffer (25 mM NaOH, 0.2 mM EDTA) individually, incubated at 95 °C for 20 min, and added to 5 μL of G2 buffer (40 mM Tris-HCl, PH = 5). The genotype of each zygote was verified by two rounds of nested PCR. The first set of primers (long section primer pair) was used to amplify a fragment approximately 500 bp in length, while a second set (short section primer pair) was designed within the first amplification product to specifically amplify either a 126 bp fragment (without HDR) or a 146 bp fragment (with HDR). The primer sequences used in nested PCR were listed in Table 1. Subsequently, gel electrophoresis was performed to identify samples with HDR, and the initial amplification products (~500 bp) from these samples were further verified using BamH1 enzyme digestion.

## Figures and Tables

**Table 1:** Summary of primer, sgRNA, target, and ssODN sequences.

Name	Nucleotide Sequence
sgRNA-T1	CTATGGAGCCGGAACCATCA
sgRNA-T2	CAGCAGCAGATTGGCAAGCA
sgRNA-T3	ACCAGTAGCCCTGATGGTTC
Target sequence in GFP reporter model	TGGGCTATGGAGCCGGAACCATCAGGGCTACTGGTCCTGCCTCCTTCTCCACTTGACCCCCAACTTTTGTTTCCTTTCCCTGCTTGCCAATCTGCTGCTG
ssODN for zygote injection	TCACAGGGCTGGCACTGCCCATGCTGCCTGGGCTATGGAGCCGGAACCATCCATAAGGATCCACGACGTCCAGCGCTACTGGTCCTGCCTCCTTCTCCACTTGACCCCCAACTTTTGTTT
Forward primer for nested PCR (long)	ACTGAGCTGTCGGCCATTCC
Reverse primer for nested PCR (long)	AGACAGCTTGGGAGCTGCAG
Forward primer for nested PCR (short)	TAGCCGGGCTGCTCACAGGG
Reverse primer for nested PCR (short)	CAAGCAGGGAAAGGAAACAA

## Data Availability

The data presented in this study are available on request from the corresponding author.
